# Supplementary material for: Waste Cooking Oils into High-Value Products: Where Is the Industry Going?
Source: Polymers (Basel). 2025 Mar 26;17(7):887. doi: 10.3390/polym17070887 (PMC11991150; doi:10.3390/polym17070887)
Supplement: Supplementary file 1 [file polymers-17-00887-s001.zip › polymers-3526922-supplementary.pdf]

SUPPLEMENTARY MATERIAL

Waste Cooking Oils into High-Value Products: Where Is the Industry Going?

Valentina Beghetto [1,2,3]

[1] Department of Molecular Sciences and Nanosystems, University Ca' Foscari of Venice, Via Torino 155, 30172 Mestre, Italy; beghetto@unive.it or valentina.beghetto@crossing-srl.com; Tel.: +39-0412348928
[2] Crossing S.r.l., Viale della Repubblica 193/b, 31100 Treviso, Italy
[3] Consorzio Interuniversitario per le Reattività Chimiche e La Catalisi (CIRCC), Via C. Ulpiani 27, 70126 Bari, Italy

Table S1. Patents regarding Waste cooking oil (WCOs) exploitation

Search criteria[(a)]	Patent N.	Assegnee	Earliest priority date	State
BL+WCO/UCO	EP1091925 A1	CARGILL	1998	Revoked Dead
BL+WCO/UCO	EP1144563 B1	CARGILL	1999	Revoked Dead
BL+WCO/UCO	WO2024206034 A1	CARGILL	2023	Pending Alive
BS+WCO/UCO	EP4150107 A1	AMPHISTAR	2020	Pending Alive
BS+WCO/UCO	EP3884054 A1	AMPHISTAR	2020	Pending Alive
BS+WCO/UCO	**KR101468070 B1**	Gno chemicals, https://www.awishkargroup.com/products/	2013	Granted Alive
BS+WCO/UCO	CN101948786B B	Petrochina, https://www.petrochina.co.id/	2010	Granted Alive
BS+WCO/UCO	CN118027688 A	HUBEI UNIVERSITY OF ARTS & SCIENCE	2024	Pending Alive
BS+WCO/UCO	CN104262561A	ZHEJIANG HENGFENG NEW MATERIAL CO LTD	2014	Revoked Dead
BS+WCO/UCO	US11767484	KING ABDULLAH UNIVERSITY OF SCIENCE & TECHNOLOGY SAUDI ARABIA	2018	Pending Alive
BS+WCO/UCO	**JP2015134896 A**	OKAMURA TEKKOSHO	2013	Lapsed Dead
PU+WCO/UCO	US8501826 B2	MALAYSIAN PALM OIL BOARD • MALAYSIAN PALM OIL BOARD MPOB	2005	Granted Alive
PU+WCO/UCO	US20160102166 A1	ADIENT LUXEMBOURG HOLDING, JOHNSON CONTROLS TECHNOLOGY BEIJING JIANGSEN AUTOMOBILE PARTS	2013	Lapsed Dead
PU+WCO/UCO	WO200453012 A1	KONKUK UNIVERSITY INDUSTRIAL COOPERATION	2002	Lapsed Dead
PU+WCO/UCO	CN107722344B B	LIU BIN • LIU LIPING	2017	Granted Alive
PU+WCO/UCO	CN106479678 A	WANG PAN • REN LIANHAI	2016	Revoked Dead
PU+WCO/UCO	EP1842866 A1	BAYER MATERIALSCIENCE	2006	Granted Alive
PU+WCO/UCO	JP2663390 B2	AGENCY OF INDUSTRIAL SCIENCE & TECHNOLOGY	1991	Expired Dead
PU+WCO/UCO	CN102206320B B	CHINA AGRICULTURAL UNIVERSITY	2011	Lapsed Dead
PU+WCO/UCO	EP3788124 A4	REG SYNTHETIC FUELS, EARL EASY SYNTHETIC FUEL SCAN LIABILITY, REG SYNTHETIC FUSE	2018	Granted Alive
PU+WCO/UCO	CN102504190B B	NANTONG HAIERMA TECHNOLOGY	2011	Granted Alive
PU+WCO/UCO	CN112625803B B	CHIA TAI INTERNATIONAL TECHNOLOGY CHANGDE GROUP	2020	Granted Alive
PU+WCO/UCO	KR101714409 B1	OILSTONES	2016	Granted Alive
PU+WCO/UCO	CN102633997B B	JIANGSU KETAI HEAT INSULATION NEW MATERIALS	2012	Lapsed Dead
PU+WCO/UCO	CN102585201B	LANGFANG HUAYU INNOVATION TECHNOLOGY & GAO CHUNQING	2011	Granted Alive

ACP+WCO/UPO	CN113845848 A	GUILIN UNIVERSITY OF TECHNOLOGY (4D printing)	2021	Lapsed Dead
ACP+WCO/UPO	AU2020102505 A4	SHENYANG UNIVERSITY OF CHEMICAL TECHNOLOGY	2020	Granted Alive
ACP+WCO/UPO	KR101848625 B1	KIM JAE CHUL	2017	Granted Alive
ACP+WCO/UPO	CN103755866B B	HUBEI UNIVERSITY	2013	Lapsed Dead
ACP+WCO/UPO	CN114854449A	GUILIN UNIVERSITY OF TECHNOLOGY	2022	Lapsed Dead
EPO+WCO/UPO	CN114854449A	GUILIN UNIVERSITY OF TECHNOLOGY	2022	Lapsed Dead
EPO+WCO/UPO	CN109082132A (B)	XI AN UNIVERSITY OF SCIENCE & TECHNOLOGY	2018	Lapsed Dead
EPO+WCO/UPO	KR20040025057 A	HWASHIN • ROAD SEAL	2002	Revoked Dead
EPO+WCO/UPO	CN105254850A	ZHENG RUI	2015	Lapsed Dead
EPO+WCO/UPO	KR100472090B1 (A)	HWASHIN • ROAD SEAL	2002	Expired Dead
EPO+WCO/UPO	CN103611468A (B)	GUANGZHOU XINCHI CHEMICAL TECHNOLOGY	2013	Granted Alive
EPO+WCO/UPO	CN103965512A	SU JUNFENG	2014	Revoked Dead
EPO+WCO/UPO	KR101848625B1	KIM JAE CHUL	2017	Granted Alive
EPO+WCO/UPO	KR20090066018A	CJNU UNIVERSITY INDUSTRY COOPERATIVE FOUNDATION	2007	Revoked Dead
EPO+WCO/UPO	KR102150710B1	HANILCON CORPRATION • CHOYANG INDUSTRIAL	2019	Granted Alive
EPO+WCO/UPO	CN109439006A	QINGDAO GUANTONG MUNICIPAL CONSTRUCTION	2018	Revoked Dead
EPO+WCO/UPO	CN103436367A (B)	JIANGSU REBO NEW MATERIAL TECHNOLOGY	2013	Granted Alive
EPO+WCO/UPO	CN110903913A	CHONGQING TECHNOLOGY & BUSINESS UNIVERSITY	2019	Lapsed Dead
EPO+WCO/UPO	CN103305347A	ARKEMA HYDROGEN PEROXIDE • JIANGNAN UNIVERSITY	2013	Lapsed Dead
EPO+WCO/UPO	CN118048046A	FUZHOU UNIVERSITY	2024	Pending Alive
APS+WCO/UCO	JP2005154467A (B2)	NIPPON OIL	2003	Expired Dead
APS+WCO/UCO	JP2005154466A (B2)	NIPPON OIL	2003	Lapsed Dead
APS+WCO/UCO	KR101714409B1	OILSTONES	2016	Granted Alive
APS+WCO/UCO	CN109266026A	CENTRAL SOUTH UNIVERSITY	2018	Revoked Dead
APS+WCO/UCO	KR20210093694A	OILSTONES	2020	Pending Alive
APS+WCO/UCO	CN106674589A (B)	CHONGQING UNIVERSITY • CHONGQING PAVEMENT TECHNOLOGY	2016	Granted Alive
APS+WCO/UCO	CN106893617A	FAR EAST UNIVERSITY	2015	Lapsed Dead
APS+WCO/UCO	CN115029008A	BEIJING UNIVERSITY OF TECHNOLOGY	2022	Pending Alive
APS+WCO/UCO	CN117511240A	EAST CHINA INSTITUTE OF TECHNOLOGY	2023	Lapsed Dead
APS+WCO/UCO	CN115073925A (B)	CHANG'AN UNIVERSITY	2022	Granted Alive

Ink solvents+WCO/UCO	EP-229377 A1	Gamblin colours	1985	Lapsed Dead
Cationic dyes+WCO/UCO	US5593459 A	Gamblin colours	1994	Lapsed Dead
Polyols/PU+WCO/UCO	CN 201010138671	Wanhua Chemical Ningbo Rongwei Polyurethane Co Ltd	2010	Alive
alkyd resin+WCO/UCO	US8895689 B2	SWIMC/ Valspar Sourcing, Inc., Minneapolis, MN (US)	2006	Granted Alive
acrylic polymer+WCO/UCO	CN109096475B	CHANGZHOU GUANGHUI CHEMICAL	2018	Granted Alive
acrylic polymer+WCO/UCO	CN102702490B B	GAUNGDONG DEKANG CHEMISTRY INDUSTRY (for INKS, Adhesives)	2021	Granted Alive

(a) BS: Bio surfactant(s); BL: Bio lubricant(s), WCO: Waste cooking oil(s); UCO: used cooking oil(s); PU: polyurethane(s); ACP: acrylic polymer(s); EPO: peroxidised oil(s); APS: asphalt pavement substitute(s).
